# A Cost-Effectiveness Analysis of Screening Strategies Involving Non-Invasive Prenatal Testing for Trisomy 21

**DOI:** 10.3389/fpubh.2022.870543

**Published:** 2022-05-31

**Authors:** Shuxian Wang, Kejun Liu, Huixia Yang, Jingmei Ma

**Affiliations:** ^1^Department of Obstetrics and Gynecology, Peking University First Hospital, Beijing, China; ^2^Key Laboratory of Maternal Fetal Medicine of Gestational Diabetes Mellitus, Beijing, China; ^3^China National Health Development Research Center, Beijing, China

**Keywords:** trisomy 21 (T21), cost-effectiveness analysis, non-invasive prenatal testing (NIPT), cell-free DNA (cf-DNA), traditional triple serum screening

## Abstract

**Introduction:**

In accordance with social development, the proportion of advanced maternal age (AMA) increased and the cost of non-invasive prenatal testing (NIPT) decreased.

**Objective:**

We aimed to investigate the benefits and cost-effectiveness of NIPT as primary or contingent strategies limited to the high-risk population of trisomy 21 (T21).

**Methods:**

Referring to parameters from publications or on-site verification, a theoretical model involving 1,000,000 single pregnancies was established. We presented five screening scenarios, primary NIPT (Strategy 1), contingent NIPT after traditional triple serum screening higher than 1/300 or 1/1,000 (Strategy 2-1 or 2-2), and age-based Strategy 3. Strategy 3 was stratified, with the following options: (1) for advanced maternal age (AMA) of 40 years and more, diagnostic testing was offered, (2) for AMA of 35–39 years, NIPT was introduced, (3) if younger than 35 years of age, contingent NIPT with risk higher than 1:300 (Strategy 3-1) or 1:1,000 (Strategy 3-2) will be offered. The primary outcome was an incremental cost analysis on the baseline and alternative assumptions, taking aging society, NIPT price, and compliance into consideration. The strategy was “appropriate” when the incremental cost was less than the cost of raising one T21 child (0.215 million US$). The second outcome included total cost, cost-effect, cost-benefit analysis, and screening efficiency.

**Results:**

Strategy1 was costly, while detecting most T21. Strategy 2-1 reduced unnecessary prenatal diagnosis (PD) and was optimal in total cost, cost-effect, and cost-benefit analysis, nevertheless, T21 detection was the least. Strategy 3 induced most of the PD procedures. Then, setting Strategy2-1 as a baseline for incremental cost analysis, Strategy 3-1 was appropriate. In sensitivity analysis, when the NIPT price was lower than 47 US$, Strategy 1 was the most appropriate. In a society with more than 20% of people older than 35 years of age, the incremental cost of Strategy 3-2 was proper.

**Conclusion:**

Combined strategies involving NIPT reduced unnecessary diagnostic tests. The AMA proportion and NIPT price played critical roles in the strategic decision. The age-based strategy was optimal in incremental cost analysis and was presented to be prominent as AMA proportion and NIPT acceptance increased. The primary NIPT was the most effective, but only at a certain price, it became the most cost-effective strategy.

## Highlights

-The proposed strategy involved NIPT primary, contingent, and age-stratified strategies in one theoretical model using parameters from the real world, which included route screening scenarios and verified current guidelines.
-Further discussion on the appropriate threshold and refining the aging group enabled us to elaborate on the policy for NIPT integration.
-In sensitivity analysis, the focus on the tendency of incremental costs enabled us to target the most influential factor, find the turning point, and help to cover as many of the real-world situations in different regions as possible.
-Although we used parameters from the real world and performed a sensitivity analysis, the conclusions could be argued on the actual demographics of the population and all other real-life factors in this theoretical model.

## Introduction

Trisomy 21 (T21) is the most frequent chromosome abnormality occurring at birth (1), and is associated with developmental and neurocognitive delay. Some patients could not survive to adulthood (2). T21-related testing in routine prenatal care is available in most countries including China (3, 4). To avoid the invasive procedure-related miscarriage, the prenatal diagnosis (PD) through chorionic villus sampling (CVS), amniocentesis, and cordocentesis is confined to high-risk pregnancies with indications of high risk after screening testing or advanced maternal age (AMA) (5). Thus, non-invasive screening with ultrasound and/or serum biomarkers is the primary strategy for all pregnancies. Traditional triple serum screening (TTSS) is the most widely used in China (6), accounting for 40% of total birth registration in 2019. As an important supplement for those missing first-trimester antenatal screening, the detection rate (DR) varies between 67 and 70%, if one fixes the false positive rate (FPR) as 3% (7).

Since 2011, fetal cell-free based non-invasive prenatal testing (NIPT) for T21 screening has been applied to clinical practice globally. The original implementation was for pregnancies with high risk, which improved the positive predictive value (PPV) to 96.7% (TRIDENT study) (8). Inconsistent with this, the International Society of Ultrasound in Obstetrics and Gynecology (ISUOG) (9) and the International Society for Prenatal Diagnosis (ISPD) (10) recommend a contingent NIPT strategy after traditional serum screening. The Chinese recommendation (11) further sets the risk threshold between the upper cut-off value and 1/1,000 for contingent NIPT. Recent studies suggested that the screening efficiency in general populations is similar. The study on NIPT utility as first-tier (TRIDENT-2) found that the DR and PPV of T21 were 98 and 96%, which was comparable to or higher than expected with full evaluations (12). ISPD and the American College of Medical Genetics and Genomics (ACMG) (10, 13) later suggest that NIPT could also be the first-tier screening test for the general population. A Chinese study involving 31,515 pregnancies further supports this scenario in terms of accuracy and reliability (14). NIPT implementation reduced many unnecessary diagnostic procedures and concomitant fetal loss for those with a high risk of T21 (15). The early detection of 11 gestational weeks by primary NIPT relieves family stress and anxiety.

In modern society with a delayed reproductive age, this is the key risk factor for T21. As the “second-child policy” is applied in China, the AMA population has been increasing (16). Chinese society took maternal age into consideration (11). In 2018, the Chinese guideline for preconception care and prenatal care suggests that primary NIPT could be offered to pregnancies with a maternal age of 35~39 years (17), while PD to those over 40 years. Still, the application of strategies is variable, so further evaluations are needed in the context of delayed reproductive age and the drop in NIPT cost.

Cost-effectiveness analysis is essential for strategy evaluation in terms of public health. Primary NIPT has been shown to be less cost-effective (18–22), while integration with age stratification could be different (21, 23). Ayres AC et al. confirmed that an age-based strategy was dominant when setting “combined first-trimester screening” as the baseline (21). On the contrary, one study from Turkey found that primary NIPT was costlier in either the AMA (35 years) or non-AMA group (24). Evans et al. from the United States set “primary NIPT” as a baseline for incremental cost analysis (22). They found that the contingent strategy was more cost-effective than the age-based strategy.

Meanwhile, NIPT applications have become heterogeneous among countries or regions depending on the public health policy. The most efficient and affordable way for NIPT implementation needs to be investigated (25). We adopted NIPT primary, contingent, and age-based strategies in one theoretical model for cost-effectiveness analysis. The theoretical model included one million pregnant women to ensure enough sample size in subgroup analysis. The cut-off was set as 1/300 and 1/1,000 in the contingent strategy, which was commonly used to define serum screening with high and intermediate risk. In this age-based strategy, AMA was subdivided into over 40 years or between 35 and 39 years for different screening patterns in terms of T21 incidence risks and potential harms of invasive diagnosis. The comparisons were further verified with sensitivity analysis, in accordance with increasing AMA and decreasing NIPT price.

## Materials and Methods

### Simulation Model

This study was exempted from ethics committee approval for the pure theoretical model involving 1,000,000 single pregnancies. The cost-effectiveness followed the decision tree analysis. During the study, the Consolidated Health Economic Evaluation Reporting Standards (CHEERS) were referred and checked (26).

**Figure 1:**
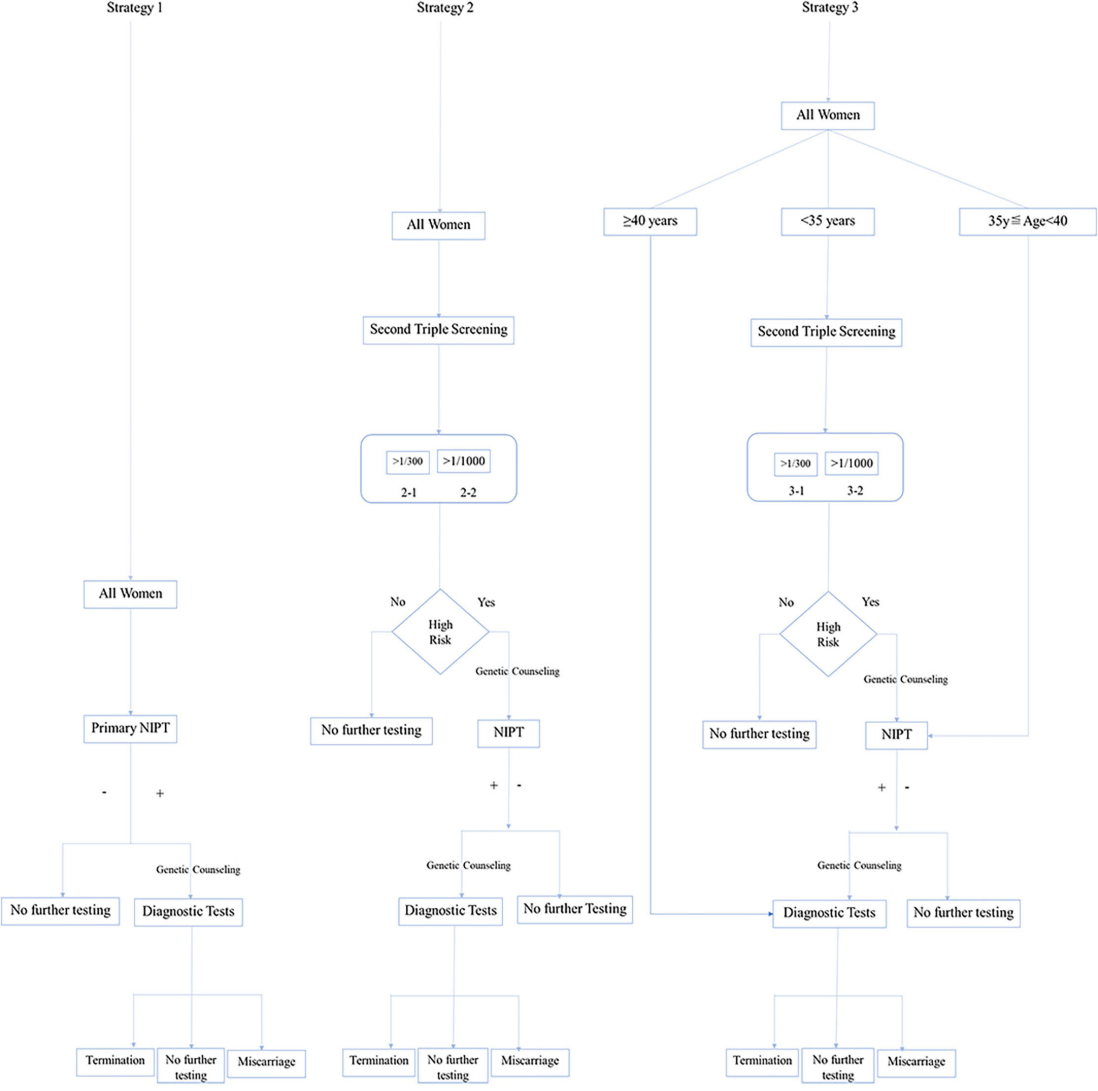
Conceptional framework of three strategies for implementing non-invasive prenatal testing (NIPT).

We presented three screening strategies (Figure 1): primary NIPT (Strategy 1), contingent NIPT after traditional triple serum screening (TTSS) as high risk (Strategy 2), and the age-based Strategy 3. Strategy 3 was stratified based on age. Invasive PD was introduced to those over 40 years old. For those between 35 and 39 years or younger than 35 years, NIPT and contingent NIPT after TTSS will be offered, respectively. The cutoff was further set as 1/300 or 1/1,000 (Strategy 2-1 or 2-2 and 3-1 or 3-2).

The incremental cost analysis was the primary outcome, in which the strategy was “appropriate” if it cost <0.215 million US$ (costs for raising one T21). The secondary outcomes included total cost, cost-effect, cost-benefit analysis, and screening performance (Detected T21, Cases for invasive diagnosis, Safety analysis, and Missed true T21) (27).

### Assumptions

The following assumptions were set for baseline analysis (21) (Table 1).

1. In each scenario, NIPT and consecutive diagnostic tests were accepted by all if needed.

2. True positive cases did not include the procedure-related miscarriages.

3. The DR and FPR of NIPT were the same in a certain population (30).

**Table 1:** Baseline and alternative values set for key variables.

**Variable**		**Baseline**	**Alternative parameters**	**Reference**
Age composition	<35	85%^a^	70–90%^*, *b*^	(16, 28)
	35–39	13%	28%-8%	
	≧40	2%	2%	
NIPT price (US$)		325^b^	<325^#, b^	
NIPT acceptance		100%^a^	30–100%^*, *a*^	(6, 29)
Invasive testing acceptance		100%^a^	90–100%^*, *a*^	(20)

*Interval analysis: The alternative parameters were analyzed at specific points at 5 or 10% intervals.

*#Continuous analysis: the alternative NIPT cost was analyzed at each point continuously*.

*a, Publication; b, on-site verification*.

### The Parameters Set

The related parameters (Table 2) were set according to the publications or on-site verification, such as age proportion (22, 28), T21 incidence in the second trimester (22, 31–33), the sensitivity (Sen) and FPR of TTSS and NIPT (7, 29, 34–36), and the incidence of procedure-related miscarriage (37).

**Table 2:** The summary of cost-effectiveness analyses related parameters.

**AMA definition**	**>35 years**		**>40 years**
Proportion based on age	<35	35–39	≧40
	85%	13%	2%
T21 incidence (1/429)	1/780	1/201	1/33
Invasive procedure related miscarriage	0.35%		
Screening method	Sen	FPR	
Cut-off value set for second serum screening	1/300	83%	8%
	1/1,000	95%	26%
NIPT	99.3%	0.2%	

Cost parameters were determined by published articles (38, 39), and nationwide and regional public data in 2019 (such as, consumer price index, CPI, and gross domestic product, GDP). The cost of T21 livebirth embraced two kinds of economic burden. The medical cost (surgery, inpatient, and recovery) and non-medical cost (transportation fees, costs for developmental service support, such as specific education, and rehabilitation exercises) generate the direct burden. The economic loss caused by patients and their parents for accompany caused an indirect burden. The exchange rate (¥–$) on the day of data processing was used to calculate 7.0729. (24 September 2019).

### The Parameter Calculation

**Figure 2:**
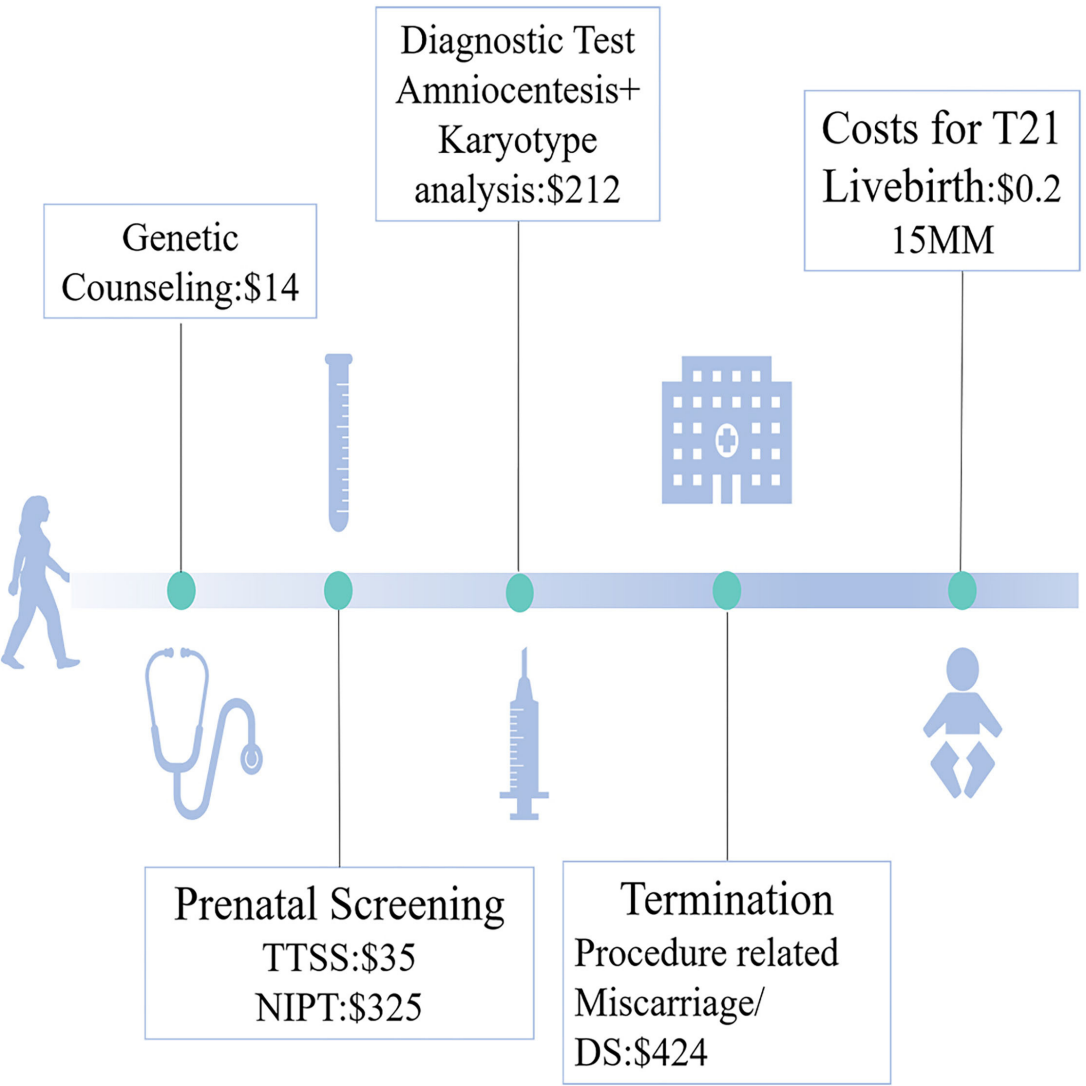
The cost parameters during the process of prenatal care for trisomy 21 (T21) detection.

The costs for prenatal tests and raising one liveborn T21 are shown in Figure 2. We calculated total costs from the social perspective (28). Both medical costs (prenatal clinical and laboratory costs, such as prenatal counseling, screening, diagnosis, and surgery for miscarriages) and postnatal costs for missed T21 livebirth were covered.

The cost-effect and incremental cost analysis were defined as medical costs for detecting one and an additional one T21 livebirth, respectively. If the cost for raising one T21 was 0.215 million US$, and if the incremental cost was less, the strategy was defined as “cost-effective.”

The cost-benefit analysis was a “benefit-to-cost” ratio, in which the “benefit” referred to the saving costs from raising one T21, and the “cost” referred to the medical costs.

Safety analysis was defined as the number of patients undergoing prenatal diagnosis for detecting one T21.

### Sensitivity Analysis

A sensitivity analysis was performed on various assumptions in the model (Table 1). The alternative assumptions took an aging society, reducing NIPT price and compliance into consideration, to figure out the most influential parameters (6, 11, 21, 25, 40). We focused on the incremental cost analysis, which reflects “effect” *via* comparisons among different scenarios and “benefit” if <0.215 million US$.

## Results

### Baseline Analysis

**Figure 3:**
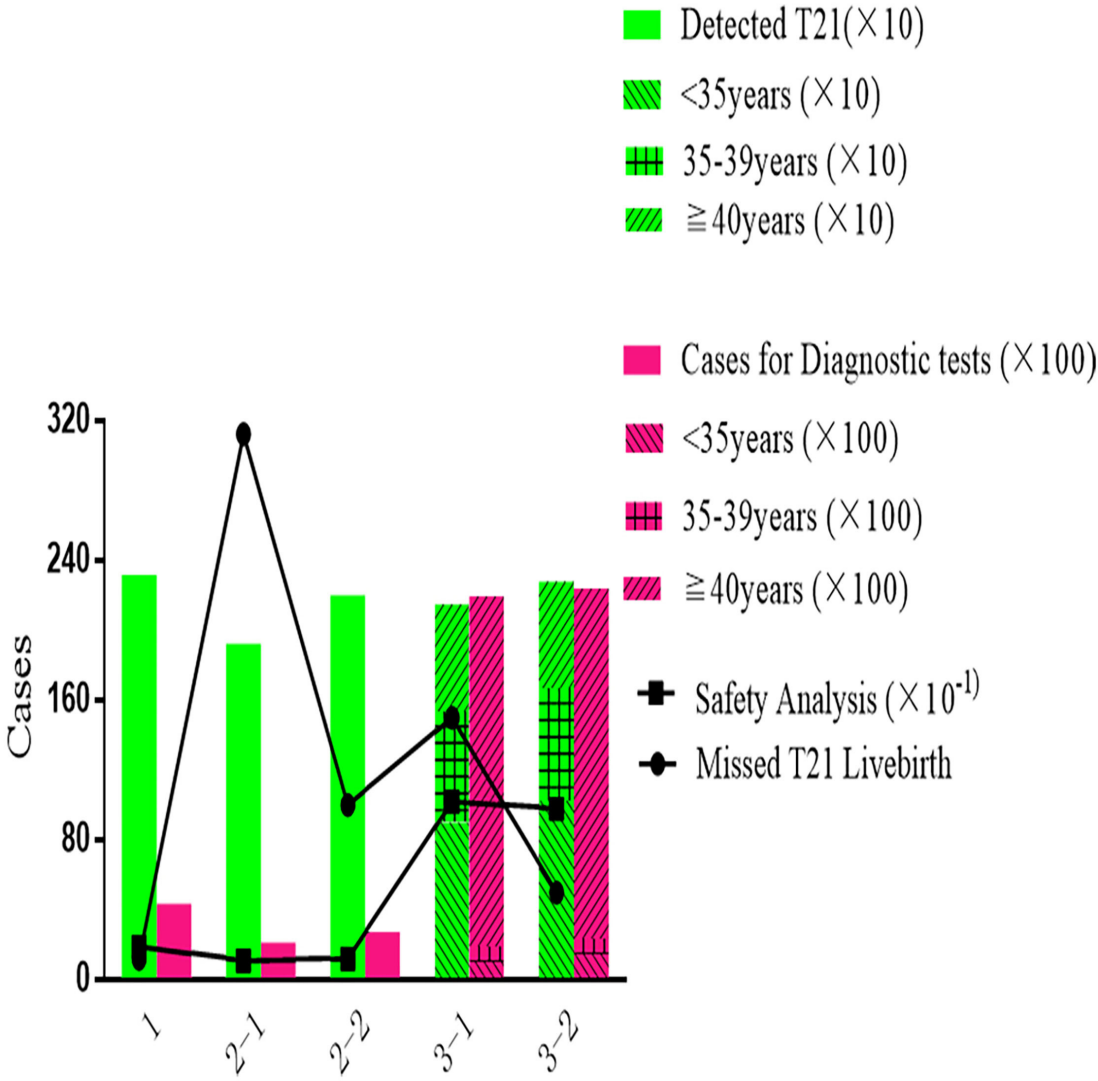
The performance and accuracy of different strategies.

As shown in Figure 3, most T21 could be detected by Primary NIPT in Strategy 1, while the Contingent Strategy 2-1 was detected at least with 312 missed cases. Being stratified by maternal age, Strategy 3 increased the DR (2,146 in 3-1 and 2,276 in 3-2) and reduced false negative (150 in 3-1 and 50 in 3-2). The price for the age-based strategy was safety analysis, which was extremely high at 10.2 (21,935/2,146) in Strategy 3-1 and 9.8 (22,370/2,276) in Strategy 3-2. With NIPT implementation, unnecessary invasive tests were significantly reduced in strategies 1 [4,310] and 2-1 (2,081). The safety analysis in primary Strategy 1, Strategy 2-1, and 2-2 was 1.9, 1.1, and 1.2.

**Figure 4:**
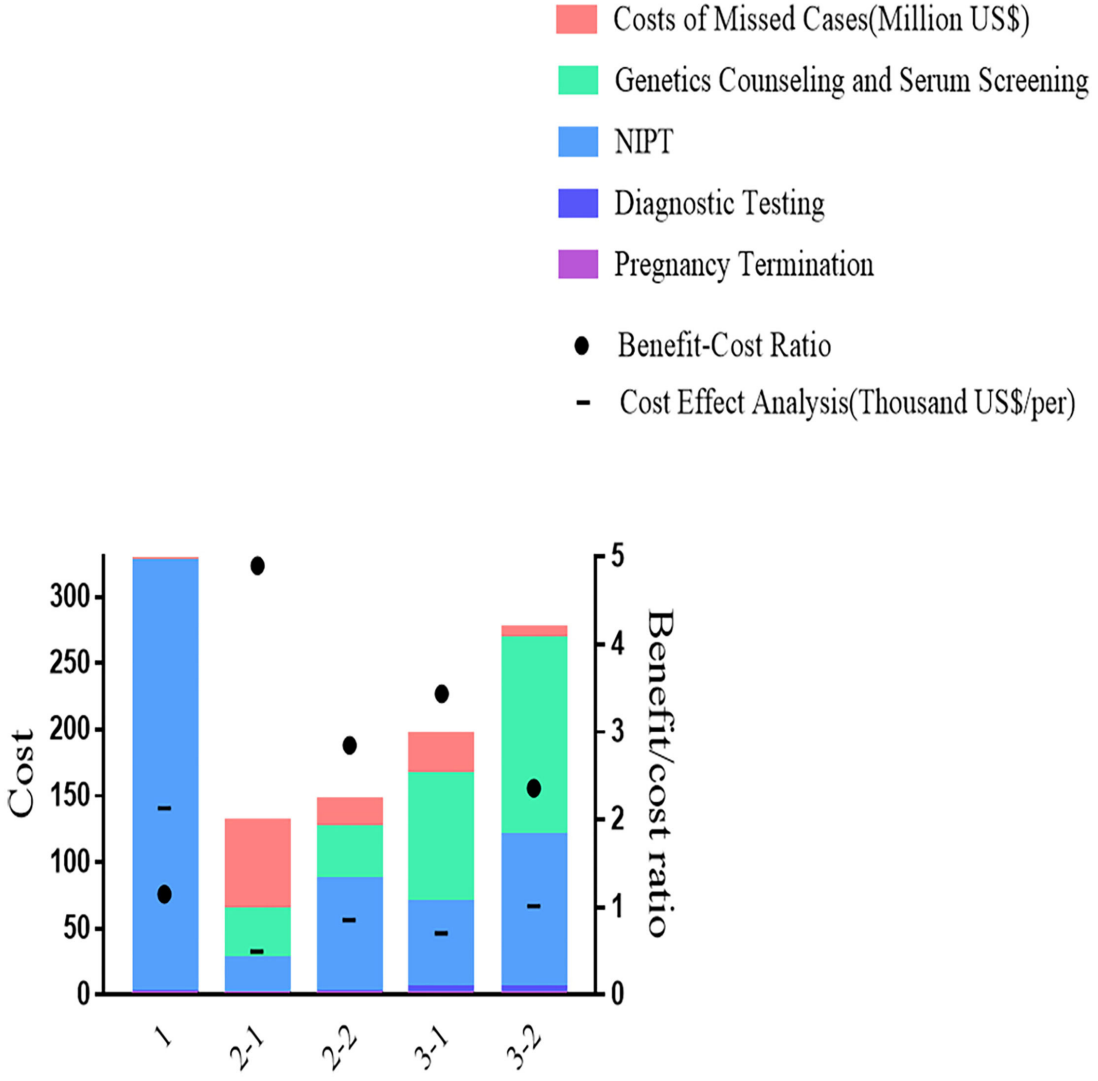
Total costs, cost-effect, and cost-benefit analysis of different strategies.

Despite having the best performance in screening efficiency, Strategy 1 was the most expensive in terms of total cost (330 million US$) and cost-effect analysis (141 thousand US$), with medical costs accounting for 99% and the remaining 1% were for missed viable cases. In Strategy 2-1, the total cost (130 million US$) and cost-effect analysis (33.4 thousand US$) were the least. The benefit-to-cost ratio in the cost-benefit analysis showed a similar pattern, as Strategy 1 was 1.16, which saved the least from the cost. The highest ratio of 4.90 was presented in Strategy 2-1. As shown in Figure 4, Strategy 3 was moderate in total cost (132 million US$ in 3-1 and 163 million US$ in 3-2), cost-effect (47 thousand US$ in 3-1 and 67 thousand US$ in 3-2), and cost-benefit analysis (3.44 in 3-1 and 2.37 in 3-2).

Setting Strategy 2-1 as the baseline for incremental cost analysis. The most incremental cost needed was Strategy 1 (0.67 million US$), followed by Strategies 3-2 (0.25 million US$) and 2-2 (0.22 million US$), all of which were inferior to Strategy 2-1. As shown in Table 3, Strategy 3-1 presented the best for the primary outcome (0.165 million US$).

**Table 3:** The incremental cost analysis setting Strategy 2-1 as baseline (Million US$).

**Assumptions**		**Strategy** **1**	**Strategy** **2-2**	**Strategy** **3-1**	**Strategy** **3-2**
Baseline alternatives		0.67:1	0.22:1	0.17:1*	0.25:1
Composition of age younger than 35 and 35–39 (16, 28)	70/28%	0.67	0.22	0.09*	0.13*
(98% in total)	75/23%			0.10*	0.15*
	80/18%			0.12*	0.18*
	85/13%			0.17*	0.25
	90/8%			0.76	0.47
NIPT acceptance (6, 41)	30%	0.67:1	0.17:1*	−0.08:1	−0.39:1
	40%	0.67:1	0.18:1*	−0.11:1	−0.74:1
	50%	0.67:1	0.18:1*	−0.17:1	−3.52:1
	60%	0.67:1	0.19:1*	−0.31:1	1.45:1
	70%	0.66:1	0.20:1*	−1.58:1	0.63:1
	80%	0.66:1	0.20:1*	0.57:1	0.41:1
	90%	0.66:1	0.21:1*	0.25:1	0.31:1
PD acceptance (19)	90%	0.74:1	0.25:1	0.14:1*	0.23:1

*The “appropriate” scenario in which incremental costs are least or lower than costs for raising one viable T21 (0.215 million US$).

### Sensitivity Analysis

We then assess the feasibility of the conclusion in the context of alternative assumptions (Table 3). First is the proportion of AMA in society. As expected, Strategy 1 was not affected. When the proportion of pregnancies younger than 35 accounted for 90%, incremental costs were more than 0.215 million US$ in all scenarios. The advantage of the age-based strategy was demonstrated as AMA increased. When the proportion of pregnancies younger than 35 was <80%, the incremental costs of Strategies 3-1 and 3-2 were further decreased to 0.12 and 0.18 million US$, respectively.

**Figure 5:**
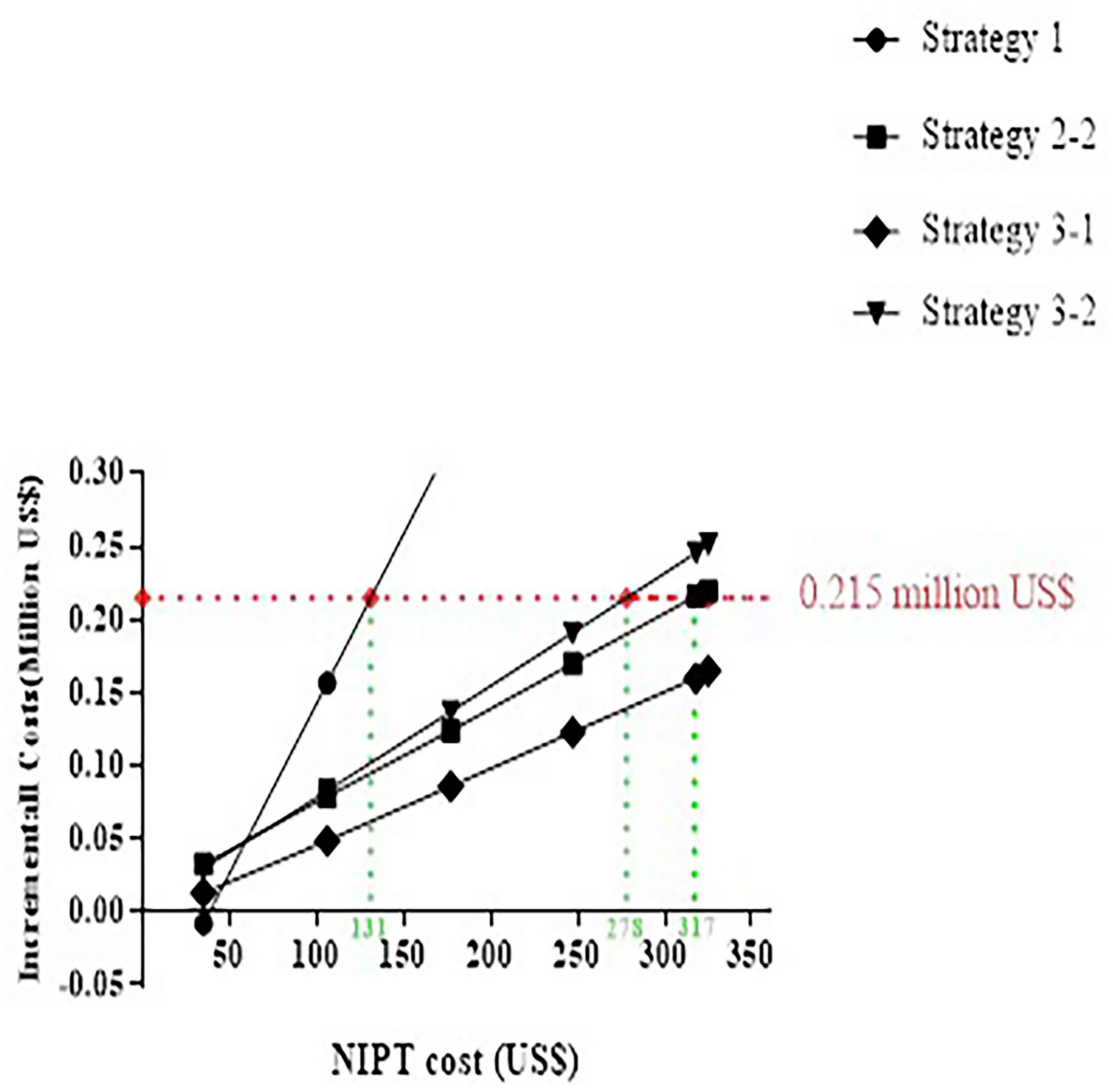
The incremental cost analysis for NIPT price-setting Strategy 2-1 as a baseline.

Primary NIPT paid a high price, although detected in most cases in the baseline analysis. As shown in Figure 5, the optimal strategy is altered along with the reduced cost of NIPT. Strategies 2-2, 3-2, and 1 became superior to 2-1 when lower than 317, 278, or 131 US$, and considered “appropriate.” If the NIPT price was lower than 47 US$, the primary NIPT reached the least incremental cost.

If NIPT acceptance decreases to 30–90%, the incremental costs ranged between 0.169 and 0.21 million US$ in Strategy 2-2, which turns to the most cost-effective strategy.

Given 90% prenatal diagnosis acceptance, the incremental costs increased in all strategies without any effect on the baseline conclusion (Table 3).

## Discussion

In the current study, the concern of primary NIPT to the general population was the cost-effective analysis. Contingent NIPT effectively reduced unnecessary invasive testing, along with the best performance in total cost, cost effect, and benefit analysis, especially in Strategy 2-1 (risk threshold as 1/300). Setting Strategy 2-1 as the baseline, the least incremental cost was the contingent NIPT strategy with age-stratified (Strategy 3-1), which was recommended by Chinese guidelines. Strategy 3-1 remained optimal in the society with an increasing proportion of AMA. The primary NIPT was demonstrated as the most cost-effectiveness option if the price dropped to 47 US$.

The parameters used in this model were rational and could represent general situations in China according to pure theoretical or population-based analysis (42, 43). Since the incidence of T21 in AMA was similar to high risk in the general population identified by serum screening (30, 44), the advantage of contingent NIPT in the current study was consistent with the previous studies (8, 19–22, 43, 45–47), from unnecessary invasive procedure reduction to total costs, as well as the cost-effect analysis. At a similar cut-off value, contingent NIPT after TTSS was much cheaper with considerable screening efficiency than direct invasive testing in an age-based strategy (15). In terms of different cut-off values in Strategy 2, lowering the threshold to 1:500 or 1:1,000 would lead to more favorable outcomes, such as more detection, but at a greater cost (15, 48). However, the appropriate threshold for contingent NIPT in the current study was different from Evans et al. study for incremental cost analysis (22), although with a similar design on age proportion and NIPT acceptance. We reached a similar conclusion for medical cost, which was 1/300. The cause could be the difference in the lower costs for raising T21 and the higher NIPT price in our system, given the fact that more cases would adopt NIPT if the cut-off were 1/1,000, which reduces false-negative cases. In sensitivity analysis, the tendency of age-stratified and contingent strategy went opposite along with the alteration of NIPT acceptance. The phenomenon further verified the influence of NIPT-related parameters on strategy implementation.

Primary NIPT has been launched as an optimal choice, especially when its price was close to traditional serum screening and lower than invasive prenatal testing (25, 49). In the current model, primary NIPT becomes dominant in incremental cost, with NIPT expense decreasing. For other scenarios, the incremental costs for NIPT were hard to be influenced, given the costs for others were far below the price of NIPT. With the development of screening technology, these conclusions were also suitable for obese pregnancies (28 ≤ body mass index (BMI) < 40), in which NIPT may not provide adequate results due to lower fetal fraction of cell-free DNA previously (50).

The current study provided a baseline for the introduction of NIPT into clinics in terms of health economics. Using parameters from the real world, the proposed strategy involving both contingent and age-stratified verified current guidelines, in which widely used clinical indications for NIPT were AMA and high risk identified by serum screening (51). Further discussion on age stratification and the appropriate threshold enables us to elaborate on the policy for NIPT integration. The sensitivity analysis of tendency considered the situations in different regions. We have identified two influential factors for policymaking, which should be taken into consideration periodically in the context of an aging society and NIPT price reduction.

Several limitations should be considered. First, this is a theoretical model taking parameters from the real world. Still, the conclusions could be argued on the actual demographics of the population and all other real-life factors. Second, T21 detection was the priority in this model considering the primary indication of PD has been the AMA in the context of delayed reproductive age, while the value of primary NIPT could extend to other genetic anomalies, such as copy number variations (15, 52). Besides, the implementation of genome-wide NIPT is under debate (12). Third, the model did not consider religious and cultural factors and merely evaluated economic costs and benefits. Notably, much more interests and benefits are at stake in case of pregnancy and screening for T21 or other forms of disability. Individual preferences and roles of healthcare providers would exert additional influences on decision-making (53, 54). Fourth, due to the absence of social investigation, the study has not taken the views of all stakeholders (health system or government). The quality-adjusted life years (QALY) is a standard and desirable indicator in cost-utility analysis (55). The area-specific judgment of individual preferences related to another person's life was the main concern, so it is not common to find QALYs in cost-effectiveness analysis on PD (56).

## Conclusions

The cost-effective analysis should be taken into full consideration during the implementation of primary NIPT. The age-based Strategy 3-1 was both effective and cost-effective in the model using parameters from China and conformed to the approved guideline.

## Data Availability Statement

The raw data supporting the conclusions of this article will be made available by the authors, without undue reservation.

## Author Contributions

SW: responsible for manuscript drafting, paper revision, and data analysis. KL: responsible for the study design and data analysis. JM: responsible for the study design, the integrity, and the accuracy of the data and analysis. HY: guarantor of this work, has full access to all the study data, and takes responsibility for the integrity of the data and the accuracy of the data analysis. All authors contributed to the article and approved the submitted version.

## Funding

National Key Technologies R&D program of China (2016YFC1000303).

## Conflict of Interest

The authors declare that the research was conducted in the absence of any commercial or financial relationships that could be construed as a potential conflict of interest.

## Publisher's Note

All claims expressed in this article are solely those of the authors and do not necessarily represent those of their affiliated organizations, or those of the publisher, the editors and the reviewers. Any product that may be evaluated in this article, or claim that may be made by its manufacturer, is not guaranteed or endorsed by the publisher.
